# Outcome and Prognostic Factors of Hemophagocytic Lymphohistiocytosis in Children: Experience From a Low- and Middle-Income Country

**DOI:** 10.7759/cureus.62494

**Published:** 2024-06-16

**Authors:** Benish Hira, Abdul Wahab Siddique, Shakeel Ahmed, Ayesha Latif, Rabiha Manzoor, Tariq Ghafoor, Awais Arshed

**Affiliations:** 1 Department of Paediatric Oncology, Combined Military Hospital, Rawalpindi, PAK; 2 Armed Forces Bone Marrow Transplant Centre, Department of Paediatrics, Combined Military Hospital, Rawalpindi, PAK

**Keywords:** survival, mortality, prognostic factors, children, hemophagocytic lymphohistiocytosis

## Abstract

Objective

Hemophagocytic lymphohistiocytosis (HLH) is a life-threatening condition especially in low- and middle-income countries (LMICs). This study was done to evaluate the outcome and prognostic factors of HLH in patients presenting to our center.

Methods

The study was carried out at the Paediatric Oncology Department of Combined Military Hospital (CMH) in Rawalpindi, Pakistan. All cases of HLH, from one month to 15 years of age enrolled between January 1, 2013 to June 30, 2023, were included. IBM SPSS Statistics for Windows, version 25.0 (released 2017, IBM Corp., Armonk, NY) was used for statistical analysis, and t-test and chi-square tests were used for comparison between continuous and categorical variables. Frequencies and percentages were calculated for categorical variables.

Results

Out of 115 patients, seven (6%) abandoned the treatment. The data of 108 cases, including 58 males (53.7%), were analyzed. The mean age at diagnosis was 31.5 ± 39.03 months. The mean time to reach a pediatric oncologist was 30.20 ± 22.15 days. Fever and pallor were common symptoms occurring in 107 (99.1%) and 98 (90.7%) cases, respectively. Jaundice was present in 44 (40.7%), visceromegaly in 64 (59.3%), and bruising/bleeding in 16 cases (14.8%). Twenty-six (24.1%) patients underwent hematopoietic stem cell transplant (HSCT), out of which 17 (65.4%) children were cured. Overall survival at two years, five years, and 10 years was 38%, 37%, and 36.1%, respectively. Disease-free survival at two years, five years, and 10 years was 33.3%, 32.4%, and 31.5%, respectively.

Conclusion

HLH leads to high mortality due to delayed or misdiagnosis in LMICs. Early diagnosis and early referral to a pediatric oncologist is the detrimental factor in survival for HLH. HSCT is the treatment of choice for primary, refractory, or relapse cases.

## Introduction

Hemophagocytic lymphohistiocytosis (HLH) is caused by immune cell dysfunction of T lymphocytes, macrophages, and natural killer cells leading to hyperinflammation [1-3]. In children, mostly inherited immune deficiencies lead to this syndrome [4]. HLH can be either inherited/primary or acquired secondary to infection, malignancy, or inflammation. Primary HLH includes familial hemophagocytic lymphohistiocytosis (FHLH) and familial erythrophagocytic lymphohistiocytosis [1,4]. Many genetic defects in primary HLH, particularly FHLH, have been recently quoted including perforin (PRF1), Munc13-(UNC13D), syntaxin 11 (STX11), and Munc18-2 (STXBP2) [1]. Viruses, specifically EBV, also serve as the incidental trigger of FHLH [5].

The Histiocytic Society in 1991 published the initial diagnostic guidelines. In 2004, these were modified by considering genetic analysis. Lately, a new analytical score, the H-score, has been used for diagnosis. The H-score significantly correlates with the HLH-2004 criteria, allowing for an effectual estimation of an individual’s risk of having HLH [6-8]. In centers where genetic testing is not obtainable, the H-score is a good auxiliary for disease classification [7].

HLH presents as a febrile illness with multi-organ involvement. Initial signs and symptoms of HLH can mimic common infections, fever of unknown origin, hepatitis, or encephalitis, making the estimation of real incidence difficult worldwide. Some reports estimate 1.2 cases per million children per year with infants being most susceptible [9-11]. HLH carries a high risk of mortality, specifically primary HLH; nonetheless, the treatment of HLH is itself associated with a high risk of morbidity and mortality [11-13]. Therefore, a high degree of suspicion must be exercised for early diagnosis, aggressive treatment, and supportive care for HLH.

This study provides an overview of children with HLH presenting to a tertiary care hospital in a resource-limited country over a duration of 10 years. Our aim was to analyze the outcome of HLH and the prognostic significance of laboratory parameters in a resource-limited country.

## Materials and methods

This prospective study was carried out at the Paediatric Oncology Department of Combined Military Hospital (CMH), Rawalpindi, Pakistan. All cases with HLH from one month to 15 years of age enrolled between January 1, 2013 and June 30, 2023 were included in the study. Patients with a clinical diagnosis of HLH but subsequently lacking follow-up data were excluded from the study. Approval from the Institutional Review Board of CMH Rawalpindi, Pakistan (serial no. 545) was acquired, and informed consent of the parents/guardians of the patients was obtained.

Detailed medical history and clinical examination was performed at the time of the first admission. Other parameters like reporting time, prior treatment details, and socioeconomic status were also recorded. The basic demographic and clinical data including age, sex, weight, surface area, temperature, pallor, bruising, bleeding, jaundice, visceromegaly, cerebral symptoms, respiratory symptoms, and lymphadenopathy were documented.

Diagnosis of HLH was made on the diagnostic HLH 2024 criteria, H score, and genetic studies. The initial workup included a full blood count, coagulation profile, and biochemical profile including hepatic and renal function tests. Bone marrow examination was performed in all patients with suspicion of HLH. The diagnosis was confirmed by genetic studies where applicable.

All patients were treated according to the treatment protocol of the Second International HLH Study 2004 modified according to local institutional guidelines. Dexamethasone and etoposide were given to all patients as initial therapy for eight weeks followed by continuation therapy of 40 weeks. Patients with CNS involvement were also given intrathecal chemotherapy. Response to treatment was assessed by lack of fever, reduction in spleen size, normal platelet count, and fibrinogen and ferritin levels.

Supportive care

At the time of diagnosis, all patients were hospitalized for initiation of HLH treatment, monitoring, and management of any complications. Electrolytes were monitored to maintain their levels within the normal range; in particular, serum potassium level was maintained >4 mmol/L, magnesium >0.75 mmol/L, and calcium level was maintained within the normal range (2.25-2.74 mmol/L). During the induction phase of the treatment, serum ferritin, triglyceride, and fibrinogen levels were tested, and the presence of visceromegaly was documented after two weeks to monitor treatment response. 

All cases of febrile neutropenia were treated with broad-spectrum intravenous antibiotics. The fever was defined as a single body temperature reading of >38°C or two readings >37.5°C at least two hours apart. Neutropenia was defined as an absolute neutrophil count (ANC) of <1000 cells per microliter. Febrile patients with ANC<1000 were treated with a combination of piperacillin-tazobactam and amikacin. Vancomycin or teicoplanin was added if central venous line infection was suspected. Piperacillin-tazobactam was replaced with meropenem if the fever continued after 48 hours. Amphotericin B was added empirically if the fever continued beyond 96 hours. Blood and blood product transfusion was given regularly. The hemoglobin transfusion threshold was 8.0 g/dL. Fresh frozen plasma, fibrinogen, and/or cryoprecipitate and platelet were transfused to maintain the fibrinogen concentration and platelet count above 100-150 mg/dL and 30-50 x 109/L, respectively.

Cases were labelled as in remission if they had an adequate clinical and biochemical response. Failure to obtain at least a partial response two weeks after standard HLH therapy was regarded as refractory HLH. Central nervous system (CNS) involvement was labeled when neurological symptoms occurred, pleocytosis and/or proteinosis in CSF were seen, or abnormalities on magnetic resonance imaging were reported. Disease-related death occurred within the first two weeks after initial presentation due to the disease itself and its complications.

Statistical analysis

IBM SPSS Statistics for Windows, version 25.0 (released 2017, IBM Corp., Armonk, NY) was used for statistical analysis, and t-test and chi-square tests were used for comparison between continuous and categorical variables. Frequencies and percentages were calculated for categorical variables. The time from the date of complete response (CR) until relapse or death was defined as disease-free survival (DFS). The time from the day of diagnosis to the day of the last follow-up or death was defined as overall survival (OS). Censoring was done at the date of the last contact (31st August 2023).

The median follow-up time was 40.90 + 45.19 months (interquartile range, 10.40-90.57). Kaplan-Meier survival curves estimated the DFS and OS and were compared using the log-rank test. The Cox proportional-hazard regression model was utilized for univariate and multivariate analysis of prognostic factors with 95% confidence intervals (95% CIs). P-values of <0.05 were considered significant.

## Results

During the study period, a total of 115 patients with pediatric HLH were registered at the Pediatric Oncology Department of CMH, Rawalpindi. Among them, seven (6%) patients left the treatment after enrolment and were excluded. Data from 108 cases, including 58 males (53.7%) and 50 females (46.3%), were analyzed. The mean age at diagnosis was 31.59 ± 39.03 months (ranging from one month to 14 years). The mean time to reach the pediatric oncologist was 30.20 ± 22.15 days (ranging from two to 92 days).

Fever and pallor were the most common presenting symptoms in 107 (99.1%) and 98 children (90.7%), respectively. Bruising/bleeding was found in 16 patients (14.8%). Physical examination revealed jaundice in 44 (40.7%) and visceromegaly in 64 (59.3%) cases. Fifty-three (49.1%) cases were neutropenic at the time of presentation. Hyperferritinemia was documented in 98 (90.7%) cases. Bone marrow aspiration was performed in 98 (90.7%) cases, and hemophagocytosis lymphohistiocytosis was evident in 86/98 (87.7%). Seventeen (15.7%) cases had Griscelli syndrome, while leishmaniasis was documented in seven (6.5%) cases. Genetic mutation of FHLH was available in only six (5.5%) cases.

Treatment outcome

**Figure 1:**
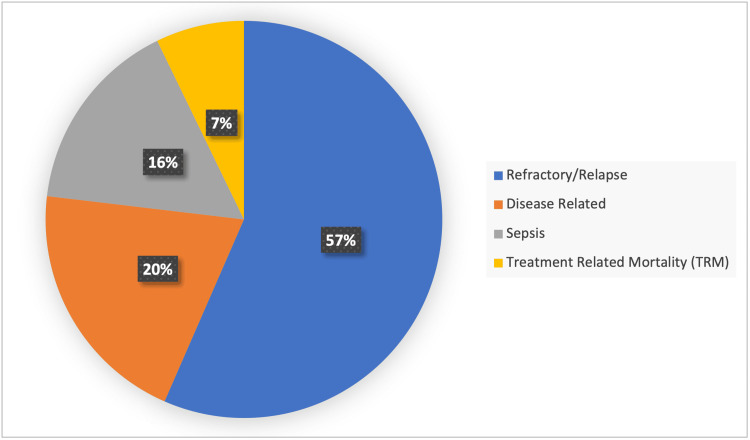
Mortality distribution of cases (n = 69)

Febrile neutropenia was the most common complication documented in 86 (79.6%) cases. Forty-one (38%) cases expired during the initial therapy phase of treatment. Out of 108 cases, 69 (63.9%) expired including 39/69 (56.5%) death due to refractory/relapsed disease, 14/69 (20.3%) disease-related deaths, 11/69 (15.9%) deaths due to sepsis, and 5/69 (7.2%) due to treatment-related mortality, as illustrated in Figure 1. Ten (4.6%) patients are still under treatment and 29 (26.9%) have been cured and on follow-up.

After the initial response and considering financial implications, 26 (24.1%) cases were planned for HSCT, out of which 17 (65.4%) children were cured, graft failure occurred in five (19.2%) children, and four (15.4%) children succumbed to treatment-related mortality.

OS and DFS

**Figure 2:**
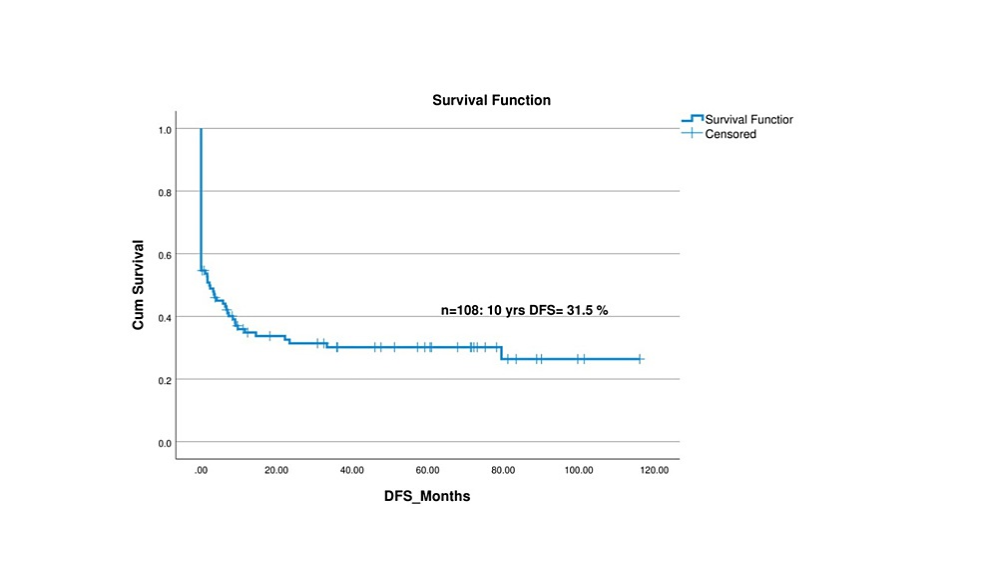
Disease-free survival (DFS) curve for children with hemophagocytic lymphohistiocytosis (HLH)

**Figure 3:**
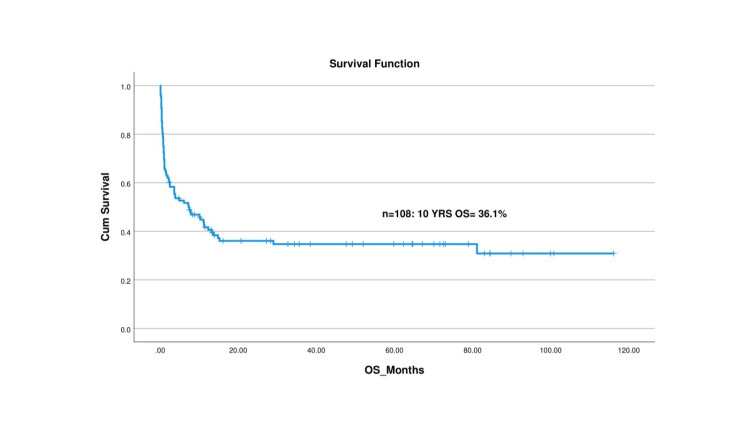
Overall survival (OS) curve for children with hemophagocytic lymphohistiocytosis (HLH) in our study

The median follow-up duration for the cohort was 49.13 ± 33.10 months. At the 10-year mark, the OS rate was observed to be 36.1%, while the DFS rate was 31.5%. A detailed analysis of the survival rates reveals that the OS rates at two, five, and 10 years were 38%, 37%, and 36.1%, respectively. This indicates a relatively stable survival rate after the initial two years, suggesting that early survival is a favorable factor for these patients. Similarly, the DFS rates at two, five, and 10 years were 33.3%, 32.4%, and 31.5%, respectively, indicating that patients who remain disease-free at the two-year mark have a relatively high likelihood of maintaining disease-free status in subsequent years. Figure 2 and Figure 3 visually represent these survival outcomes offering a graphical depiction of the Kaplan-Meier survival curves for OS and DFS. These findings emphasize the importance of early therapeutic interventions to improve long-term survival and disease-free outcomes in children with HLH.

**Figure 4:**
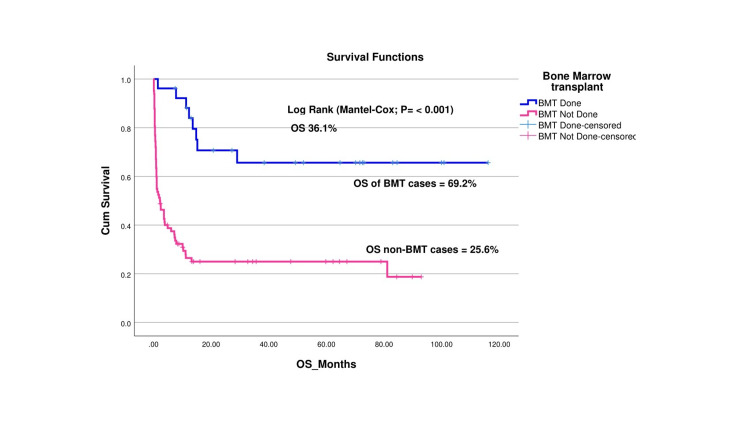
Overall survival (OS) functions of HSCT vs. non-HSCT patients BMT: bone marrow transplant, HSCT: hematopoietic stem cell transplant

The OS of patients who underwent HSCT was considerably higher compared to those who did not undergo HSCT (69.2% vs. 25.6%, respectively), as illustrated in Figure 4. This significant difference highlights the importance of HSCT in improving long-term survival outcomes for pediatric HLH patients.

## Discussion

This is the largest study on the prognosis and outcome of HLH in children from Pakistan, a low- and middle-income country (LMIC). The Pediatric Oncology Department in CMH Rawalpindi has one of the largest pediatric oncology centers in Pakistan with a patient turnover of around 350 new cases yearly.

The male-to-female ratio in our study was 1.16:1, which is in congruence with previous studies quoting ratios of 1.8:1 and 1.30 [8,14]. Other studies by Janka et al. and Mottaghipisheh et al. have also documented a male preponderance, although no clear association has been verified [8,10]. The mean age at diagnosis was 31.59 ± 39.03 months in our study, which coincides with other studies by Zhou et al. and Bayram et al. who observed a mean age of 25 and 30 months, respectively [15,16]. By contrast, another study from Iran by Mottaghipisheh et al. reported 11.5 months as mean age of presentation [8]. FHLH was observed in only 5.5% of cases as all patients were not screened keeping in view the limitation of resources and huge financial burden of genetic testing. This number is quite higher in previous literature with Alsohime et al. reporting 43% of cases of FHLH and 23.5% cases reported by Paul et al. [3,17].

Seventeen children (15.7%) were diagnosed cases of Griscelli syndrome, which is a known association observed in previous literature. Sasan et al. observed Griscelli syndrome in 12% of cases [14]. Twenty (33.3%) cases in our study had visceral leishmaniasis (VL-HLH)-associated HLH. An Indian study by Paul et al. reported 2.1% cases of VL-HLH, whereas Gamero et al. reported 41% cases, which reflects endemicity of visceral leishmaniasis in these specific regions [3,18].

Fever and pallor were observed in 90% of children in our study, which is a well-reported phenomenon in previous literature. Mottaghipisheh et al, and Shazia et al, reported fever in nearly all of their patients, while Yong-hai Zhou et al. reported a figure of 98.7% [8,15,19]. However, Alsohime et al. reported fever in only 27.6% of patients, which was in contrast to our results [17]. Bruising was present in 14.8% of children in our study, which is in accordance with previous literature as narrated by Alsohime et al. who reported bleeding in 13.8% [17]. Forty-four (40.7%) children in our study had jaundice, and 64 (59.2%) children had visceromegaly on initial presentation. Zhou et al. also reported hepatomegaly in 95.6%, splenomegaly in 92.1%, and jaundice in 34.5% of patients [15]. Similarly, Mottaghipisheh et al. also reported splenomegaly as the most constant presenting sign detected in nearly all their patients [8].

Hyperferritinemia was the hallmark biochemical abnormality observed in 90.7% of cases in our study, which has been observed in many previous studies as well. Allen CE et al. revealed that ferritin level >10,000 stands at 90% sensitivity and 96% specificity for HLH [20]. Similarly, a study by Shazia et al. from Pakistan also found increased serum ferritin levels in 82% of their patients [20].

Contrary to previous literature, we found children who were cured to have higher mean ferritin levels as compared to those who expired. However, this relationship was not significant (p = 0.5). No other study in our knowledge has observed this peculiar association. Mottaghipisheh et al. and Paul et al. did not observe hyperferritinemia correlation with disease severity or adverse outcomes, such as relapse or death [3,8].

Mean bilirubin levels in our study were 16.7 (SD = 12.4) in treated patients as compared to 41.3 (SD = 58.3) in children who expired (p < 0.05). This effect has been studied in the past and reported in the literature by several studies that total bilirubin twice the upper limit of normal is associated with a higher mortality rate [17,21].

Rungrojjananon et al. reported that platelet count <50,000 cells/mm^3^, hyperbilirubinemia, and treatment response at weeks two and eight after treatment initiation were associated with higher mortality although we did not observe these associations in our study [22]. However, we found that a higher triglyceride level was associated with inferior outcomes, with survivors having a triglyceride level of 4.04 (1.78%) as compared to nonsurvivors with a level of 7.00 (5.91%; p < 0.05). Mottaghipisheh et al. also reported this similar association, contrary to the study reported by Alsohime et al. who reported high mean triglyceride levels in survivors of HLH [8,17].

We did not observe any prognostic association between age, gender, cytopenias, albumin levels, and fibrinogen levels and the outcome of the child, as shown in Table 1. Ramchandar et al. also did not observe any association similar to our results [23]. However, Abbasi et al. reported a significant association between these parameters and the outcome of the child [7].

**Table 1:** Comparison of prognostic factors among survivors and non-survivors in HLH A p-value of <0.05 was considered significant. ANC: absolute neutrophil count, WBC: white blood cell, PT: prothrombin time, PTTK: partial thromboplastin time kaolin, ALT: alanine transaminase

Demographic and laboratory parameters	Survivors mean (SD)	Non-survivors mean (SD)	p-value
Age	42.3 (45.2)	27.8 (37.5)	0.1
ANC	2.6 (5.8)	1.4 (1.5)	0.14
WBC	6.6 (7.0)	5.1 (3.3)	0.15
Biliirubin	16.7 (12.4)	41.3 (58.3)	0.03
Hemoglobin	7.5 (1.7)	8.0 (2.1)	0.33
Platelets	90.4 (97.4)	61.1 (89.6)	0.15
Albumin	31.9 (5.1)	31.9 (6.9)	0.98
Ferritin	16989 (30324)	12878 (26160)	0.50
Triglycerides	4.0 (1.78)	7.0 (5.91)	0.04
PT	16.79 (3.6)	18.53 (11.3)	0.5
PTTK	35.7 (8.6)	39.1 (12.0)	0.26
Fibrinogen	198.5 (118.1)	178.5 (87.9)	0.39
ALT	56.3 (41.4)	79.5 (101.9)	0.26

HLH is a life-threatening disease for children with studies reporting poor prognosis without treatment and has a median survival of one to two months [24]. A study conducted by Japanese scholars found that outcomes in children with HLH who were treated with the same protocol were different among HLH subtypes [15,25]. Unfortunately, 69 (63.9%) children eventually expired in our study, which is considerably higher than data from the developed countries. Previous studies have stated a lower mortality rate ranging from 13.8% to 28.1% of pediatric HLH [15,26]. Allen et al. and Abbasi et al. also observed a higher mortality percentage of 32% and 44%, respectively [7,20]. This is mainly due to the delayed presentation of patients to oncologists and limited resources in LMICs. In addition, malnutrition is also a common feature in children from LMIC further increasing mortality risk. The differences observed in survival and prognostic factors in various studies may be due to geographical differences in patient population, different protocols used in various centers, and the lack of high-end medical care in LMICs.

Thirty-two (29.6%) children in our study expired within 30 days of disease onset, whereas 41 (37.9%) children died during the initial therapy induction phase, which is similar to previous literature by Zhang et al. and Zhou et al. who observed 30-day mortality as 30.9% and 29.52%, respectively [15,27]. Tan et al. reported that the pooled mortality rate was 32.6% (95% CI: 23.4-42.4) and was higher in primary in comparison to secondary HLH [28].

We found a peculiar observation that children presenting with secondary HLH due to visceral leishmaniasis had a better survival rate than the rest of the cases. Mottaghipisheh et al. also reported a higher OS of three years in VL-associated HLH (p < 0.001) [8].

The OS rate in our study was 37.91% at two years, 37.1% at five years, and 36.1% at 10 years, whereas the DFS was 33.3% at two years, 32.4% at five years, and 31.5% at 10 years. Mottaghipisheh et al. and Tanya et al. also reported high mortality of the primary HLH diseases with OS of three years at 35.9% and 21.89%, respectively [8,24]. Messina et al. narrated the five-year probabilities of OS and event-free survival (EFS) as 71% and 60%, respectively [29]. Moreover, one study by Ponnett et al. reported that the survival rate of HLH improved from a one-year survival rate of less than 5% to a five-year survival rate of 21% [24].

HLH has a high risk of relapse in the pediatric population. We documented a high relapse rate of 56.5% of the total cohort. Half of the children presenting with relapse eventually expired due to disease-related complications or refractory disease. In another cross-border study by Ponnatt et al., a 13% rate of relapse/refractory HLH was reported [24].

Twenty-six children in our study underwent HSCT, out of which four (15.4%) children expired due to transplant-related mortality and 17 (65.4%) children were cured. Graft failure was seen in five (19.2%) children. Allen et al. reported a post-HSCT mortality rate of 35% in his study [20]. Similarly, in a study by Messina et al., 26 cases (24%) died due to transplant-related causes, whereas 14 (13%) and 10 (9%) patients experienced graft rejection and/or relapse, respectively. Twelve out of 14 children were given a second HSCT after graft failure/relapse and were reported alive and disease-free [29].

One significant limitation of this study on HLH conducted in an LMIC is the lack of comprehensive genetic studies, particularly for FHLH. Due to constrained resources and limited access to advanced diagnostic technologies, genetic testing to identify specific mutations associated with FHLH was only performed in six cases.

## Conclusions

HLH is a rapidly progressive disease that leads to a high mortality rate due to delayed or misdiagnosis in resource-limited regions where it can masquerade initially with varied clinical presentations. This study provides valuable insights into the long-term outcomes of pediatric patients with HLH in an LMIC. Delayed presentation to a pediatric oncologist and higher triglyceride levels at the time of presentation were associated with poor outcomes. The 10-year OS and DFS rates were 36.1% and 31.5%, respectively, with OS and DFS showing significant stability after the initial two years.

Another important observation was the markedly higher OS in patients who underwent HSCT, highlighting the importance of incorporating HSCT into treatment protocols where feasible. Awareness of primary care pediatricians for an early referral to a pediatric oncologist is required as it will improve the outcome and chances of survival. Overall, this study emphasizes the importance of HSCT in the management of HLH and calls for enhanced genetic diagnostic facilities in LMICs to better understand and treat this complex condition.
